# Bicultural peace pedagogy: opportunities and obstacles

**DOI:** 10.3389/frma.2024.1469377

**Published:** 2024-11-14

**Authors:** Katerina Standish

**Affiliations:** Global and International Studies, University of Northern British Columbia, Prince George, BC, Canada

**Keywords:** peace pedagogy, Indigenous pedagogy, peace education, bicultural education, Indigenous peace education

## Abstract

This article appreciates decolonization in education, positing bicultural pedagogy as peace pedagogy. It will encapsulate peace education, peace pedagogy, colonization, Indigenous rediscovery, and Indigenization of the curriculum (biculturalism) and then turn to the transformative practice of decolonization in education. The paper seeks to propose a conceptual bridging facet from five core Māori values: *wairuatanga, manaakitanga, kotahitanga, whanaungatanga*, and *rangatiratanga*, to Indigenous pedagogy and, finally, to peace pedagogy. The alignment of Indigenous pedagogy and peace pedagogy is an attempt to evaluate the potential of bicultural peace pedagogy as a decolonizing education. The paper finds congruence between Western peace pedagogy and several gaps related to practice and cultural goals. To assist other non-Indigenous knowledge workers (termed Pākehā in Aotearoa/New Zealand) in decolonizing education, this paper has sought to elevate aspects of peace culture that align with Indigenous practices/values.

## Introduction

Peace education is broadly considered an act of learning that leads to transforming violence. Peace Education can be separated by form and audience to focus on aspects of, for example, Human Rights, Education for Sustainability, International Education, Education for Conflict Resolution and Development Education (Harris and Morrison, 2013), but also targeted educational intervention including Democratic Education (Dewey, 1916), Non-violence Education (Rosenberg, 2003), Multicultural/Citizenship Education (Banks, 2007), Global Education (Hicks, 2003), Moral Education (Carmody and Carmody, 1988; Noddings, 2002), Environmental Education (Carson, 1962), Critical Peace Education (Bajaj, 2008a; Brantmeier, 2011; Kincheloe), and Yogic Peace Education (Standish and Joyce, 2018). As each form of violence requires a unique form of intervention, peace education as a field seeks to be both diverse and responsive.

Harris (2004) and Harris and Morrison (2013) consider peace education as a form of peacebuilding—an organized “peacelearning” intervention that enhances the possibility for personal and social transformation. For Harris, and Harris and Morrison, peace education is a *personal* activity leading to non-violent social change and environmental sustainability. Reardon (1988) defines peace education as tools and content that work toward the creation of comprehensive peace—peace in *person* and *society* while Freire considers peace education to be about perceiving *personal* and *social* reality to act on and transform such realities via “critical consciousness” (Freire, 2003). Whereas, Harris, and Harris and Morrison consider the “work” of peace education to be individually transformative—where individuals are each equally enabled and empowered through the theories, philosophies and practices of peace education—Reardon and Freire consider social transformation (not personal transformation) to be the ultimate goal, one that leads us toward gains of universal equality.

However, Western concepts of universalism and equality are challenged by particularisms that recognize distinction as key. For example, Trifonas and Wright (2013) problematize notions of “equity” through an appreciation of “difference” to engage with ideological biases and discriminations amongst different communities—particularly in Western education. Where the “work” of peace education is *individual* for Harris and Harris and Morrison and *societal* for Reardon and Freire, Trifonas, and Wright alert us to the role of peace and education *in institutions* and consider the role of peace education to be a pedagogical praxis that must engage with negative history, troubled pasts, difficult information, and problematic cultural narratives.

The platform of understanding evident in Harris and Harris and Morrison's consideration of peace education and that considered by Reardon, Freire, and Trifonas and Wright reveals a tension of “intention” where the focus of peace education is formerly the actualization of individuals who can then work toward interpersonal and social transformation vs. the creation of an externally focused ethics of equanimity that identifies schools and education systems as onto-epistemological constructs of subjectivity. Simply put, using peace education without first deconstructing the underlying assumptions present in notions of learning and learning spaces means leaving unexamined the challenges individuals have working toward peace (non-violence) in *already* culturally violent spaces. In exploring the possibility of a “bicultural peace pedagogy,” this tension must be abundantly appreciated. This article offers an appreciation of decolonization in education, positing a bicultural pedagogy as peace pedagogy.

### Peace pedagogy

Pedagogy refers to the actions of teaching and the purpose and delivery of educational materials. The purpose of “peace” pedagogy is to articulate the act of teaching to the aim of humanization via actions of emancipation and non-violence. Several contemporary scholars/practitioners articulate what can be considered “peace” pedagogy in the following manner: to Harris, peace pedagogy is a way to usher in peace via five peace techniques: “dialogue, cooperation, problem-solving, affirmation, and democratic boundary setting” (1990, p. 255). To Jenkins (2015), peace pedagogy is “the antithesis of indoctrination…an ethical, elicitive, and learner-centered approach to worldview transformation that honors the dignity and subjectivity of the learner” (p. 3). The International Institute of Peace Education (IIPE) sources peace pedagogy in transformative education, “with roots in Dewey, Freire, and Montessori…[placing] emphasis on helping learners to think critically…,” based on student-led and self-directed acts of learning (cited in Jenkins, 2008, p. 168).

Pedagogies of peace are both techniques and objectives, aims that alter landscapes of violence via Western educational techniques of cognitive growth (Piaget, 1965), ecological learning (Bronfenbrenner, 1979) and relational development (Vygotsky, 1981) but also methods of critical learning taught to foster equality and social justice (Kincheloe, 2008), and utilizing pedagogies of critical empowerment (Giroux, 1988). Although Harris averred that “peace education is not attractive to social activists who want to confront structural inequalities” (Harris, 2004, p. 8), other theorists perceive peace education and peace pedagogy as vital to creating social change through education. Reardon (1988) considered education vital to “changing the social structures and patterns of thought that have created them” (p. x), Synott (2005) amplified acts of education that challenge “dominant models of education that reproduce oppressive, violent social structures” (p. 7) and Bajaj (2008b) considers a major unifying facet of peace educators “optimism that education can lead to positive social change” (p. 3).

Harris characterized mainstream Western education as a construct of dominatory and competitive force resulting in passivity and powerlessness (Harris, 1990). He argued that teachers must first understand their role and practices that uphold these violence(s) to contribute to altering them. In the clearest definition of “peace pedagogy” found in the literature, Harris describes five pedagogies that counter domination, competition, force, and passive powerlessness in the classroom:

(1) The use of *dialogue* promotes a perspective that students and teachers have important insights into the truth. (2) A *cooperative class* breaks down competitive procedures that can contribute to structural violence. (3) *Problem-solving* teaches students how to become active learners and generate solutions for problems in the classroom rather than passively digesting information. (4) *Affirmation* builds student confidence to counteract feelings of powerless[ness]. (5) *Democratic boundary setting* involves students in setting classroom rules so that teachers do not have to use force to control their pupils (Harris, 1990, p. 260).

Such peace pedagogies are subsequently enlarged to include acts focusing on “cooperation, sharing, participation and fellowship” (Jenkins, 2008, p. 169). As the focus on peace pedagogy moved from the role of teachers to students' experience, the aim of “relational” peace pedagogy moved from technique to objective. Here, Standish considers peace pedagogy more than community building but also propagative of “futures thinking,” where positive socialization in peace education leads to more than ideas about what needs to change in society but also pathways thinking and agency to do so (Standish, 2018). Building upon hooks (1994) notion of transformative pedagogy, Standish and Joyce (2018) advocate for building individual esteem and wellbeing as pedagogies of peace and that while social change is an archetypal outcome of peace education, hooks and Standish and Joyce advocate for pedagogies that do more than transfer content to students that lead to social transformation but also pedagogies that support students' self-actualization and humanization—both necessary for facing structural social violence. Through what Standish and Joyce describe as a pedagogy of “self-knowledge” and what hooks termed “engaged pedagogy” the role of teaching is not simply information transfer but wellbeing and wellness—an affective and cognitive result of humanization.

In summary, while Harris conceptualizes practices of the community as generative of a peaceful classroom and Reardon, Synott, Bajaj, Kincheloe, and Giroux consider education a conduit for social change, to Jenkins, hooks, and Standish and Joyce, the formation of community via practices of positive relationship generate humanization.

### Colonization

The beginning of the Indigenous and non-Indigenous world as a cultural fulcrum of identity is traced to the global phenomenon of colonization—a social, economic and political process of European Imperial domination and subjugation that began roughly 500 years ago (Betts, 2012; Bell, 2004; Hokowhitu, 2010). The advent of colonization created a category of identity that collapsed diverse and dissimilar peoples into a binary of either the “prior” occupants of lands or the invaders/settlers (Bell, 2004). As the clock of Indigeneity begins at European contact during a global Imperial conquest that involved “the ‘power' of a people to ‘reproduce' itself in different spaces,” this reproduction “produced” new forms of being and identity (Ferro, 1997, p. 11).

While colonization is often considered something in the past, the effects of colonization are far from historical as the “social process” of cultural and material values still center upon European/Western economies and epistemologies (Trask, 1993; Gandhi, 1998; Laenui, 2000, p. 150). Despite the postcolonial moment, an ongoing “coloniality” **(**Quijano, 2000) is experienced within so-called postcolonial nations such as Aotearoa/New Zealand, where hierarchies (racial, gendered and economic) that were established by colonial powers endure. The hierarchy places the European (the white, male, wealthy European) above all others and justifies dominance based on racialized categories of constructed biological inferiority. Despite multiple forms of agency and activism, the bottom rung of such hierarchies of humanity has been occupied by Indigenous Peoples the world over since colonization, as their cultures and their very humanities have been fragmented and destroyed to make way for settler colonial culture (Smith, 2012).

### Indigenous rediscovery

The decolonial process, whereby previously colonized territories and peoples begin to unravel and divest from colonial structures of power (Betts, 2012), involves processes of recognition and reclamation that include aspects of Indigenous rediscovery (Laenui, 2000). In this rediscovery, Indigenous Peoples have come to see how their very “definition” as humans was the result of colonial “naming” and “quantifying” strategies designed to eradicate and erase them (Smith, 2012; Arnold, 2018). This rediscovery has led to several differing categorizations of Indigenousness that foreground “sacred histories, ceremonial cycles, language, and ancestral homelands” (Alfred and Corntassel, 2005, p. 609), legislated identities based on blood quantum (Smith, 2012), as well as “imagined” constructs of Indigenousness (Leoni et al., 2018) forged in opposition to settler identity (Weaver, 2001). The definition of Indigenous Peoples that I subscribe to is the definition affirmed by Maaka and Fleras (2005):

Indigenous peoples are those who occupied their lands prior to European discovery and settlement and continue to do so; whose descendants can trace some degree of historical continuity from the past to the present; who have retained social and cultural differences that are distinct from the other segments of the population; and who remain marginalized as a colonized enclave (p. 30).

## Biculturalism: indigenization of the curriculum

In Māori—the predominant indigenous language in Aotearoa/New Zealand, *Te Rangahau Māori* means undertaking research that increases understanding of mātauranga Māori, and *Ngā Whakahaerenga Pai* seeks to produce and develop learning and teaching environments to engage with mātauranga Māori. Indigenous Māori and non-Indigenous Pākehā comprise the two groups—the bicultures—of Aotearoa/New Zealand. Fashioning a platform of understanding regarding bicultural culture and education from a non-Indigenous (Pākehā) perspective is a trial facing Indigenous-Settler Indigenization worldwide. To do so from an Undigenous vantage point, is more challenging still. Undigenous refers to being both non-Indigenous but also non-native born: “a double outsider, not native…and not Aboriginal either;” as global migration reconstitutes so-called “settler” descendants, the cultural norms of education will need to check their tethers (Standish, 2019. p. 126).

In bicultural initiatives, the expectation (explicit or otherwise) is that the curriculum will be indigenized. The reality for many with “double outsider” status is that there seems to be little direction on exactly what this means, or how this can/should/must be done. Though I share with many of my colleagues a desire to contribute to decolonial education initiatives, I also feel that many of us feel we are on our own.

My decolonization began in 2016 when I started to read in earnest literature regarding Canada's decolonizing education movement. There is a unique and gruesome connectivity of education in the Native Genocide of Indigenous peoples in Canada, an official component of the Indian ACT actioned in residential schools. Children were taken from their homes, and many did not ever return. Those who endured faced daily aggression, violence and terror. The ability of the Canadian government to acknowledge this heinous history (the last Indian Residential school was closed in 1996) and commit to decolonizing the Canadian education system (systems which are provincial, not federal) is a reverie of conceptual instability, and inconsistent implementation.

As a Canadian academic working in Aotearoa/New Zealand, I asked myself “How can I be an instrument of this important change without first understanding this?” Sadly, there is no handbook for *decolonization* for the non-Indigenous nor guide to *Indigenization* (two distinct initiatives, with different agency and audiences). As no journey into decolonization in Aotearoa/New Zealand could ensue without understanding the past, I sought information to explain what biculturalism means.

In the New Zealand context, biculturalism refers to the conceptualization of two ethnically and culturally different peoples (Māori and Pākehā/European) in a social and political partnership relationship. This relationship was established when representatives of each group signed the Treaty of Waitangi in 1840. The Fourth Labor Government first developed bicultural policies elected in 1984. It was an acknowledgment of, and response to, the historical injustices suffered by Māori people as a consequence of colonization…[and] marked a shift from previously monocultural government policies which included at various times a mixture of assimilation, integration and separatism of Māori people (Lourie, 2016, p. 638).

Although policy, biculturalism did not immediately gain significant ingress. James Ritchie compiled a guide for Pākehā to understand and behave appropriately in Aotearoa/New Zealand titled “Becoming Bicultural.” He proclaimed (almost 30 years ago),

We must fashion the bicultural world of inter-connections and common pathways and understandings, but we will not be successful in this until the Māori world is respected, is resourced, is in good health and strength, and is in a true state of equity (1992, p. 11).

For Ritchie, the drive for biculturalism was not about simply recognizing and then arguing about an ancient treaty but the need to forward bicultural identity, which means “Māori assertiveness and Pākehā acceptance;” for a cultural change from the dominant “white” settler society to one that fully embraces Indigenous validity and therefore cultural expression and ideology (1992, p. 202). In terms of how this affects education, Ritchie opined that a parallel education system divided by race/ethnicity was unlikely and that as the reality was that “most children and young people will continue to receive their education in the general system… [the general system] must become more and more Māori” (p. 202).

The bicultural initiative was a result of the deliberate institutionalization of Aotearoa/New Zealand's two ethnic groups in education and a recognition of social and racial experiences of disadvantage resulting from the widespread ethnic supremacy of Pākehā (Belich, 2001; Lourie, 2016).

Bicultural educational policy in New Zealand is partly a response to the ongoing challenge of educational underachievement of Māori students in the compulsory schooling sector. [As] the dominant bicultural discourse frames the educational underachievement of Māori as a problem associated with cultural differences, Bicultural education policy is thought to be a means of addressing the ongoing challenge of educational underachievement of Māori students in the compulsory schooling sector (Lourie, 2016, p. 637–8).

Bicultural education in the general education system is an initiative that has unpredictable and variant levels of implementation, advocacy, internalization and integration through Aotearoa/New Zealand, but for the purposes of this review it simply means that in colonial spaces that absented Indigenous peoples and erased points of view and practices already present in this Aotearoa/New Zealand, biculturalism is both an understanding of first principles of the country (based as it was on the Titriti o Waitangi/Treaty of Waitangi), but also the recognition that as schools are major sites of secondary socialization that impart values and ideals, cultural constructs and notions of both knowledge and responsibility to the young, the Western education model that characterizes Aotearoa/New Zealand could not remain monocultural, racist, dominatory, and discriminatory against Māori (Bronfenbrenner, 1979). While Bishop (2012) analysis of the implementation of the Te Kotahitanga Effective Teaching Profile in Aotearoa/New Zealand showed “participation, engagement, retention, and achievement of Māori students in Te Kotahitanga schools are improving compared to a comparison group of schools,” mainstream education, in non-Te Kotahitanga schools was still needed (p. 42). As bicultural education aims to recognize and elevate the social, economic and cultural relevance of being Māori in education, it needs to be a practice of non-Indigenous knowledge workers, too.

The original goal of biculturalism was the inclusion of a Maori culture that had been marginalized and maligned in Pākehā culture. Although the scope of this article does not permit me the space of a detailed history of biculturalism or bicultural education in Aotearoa/New Zealand, including Māori medium learning and or assessment, evaluation or critique of the success or failure of bicultural education initiatives, I would like to present a rationale for seeking to identify the potential of a “bicultural peace pedagogy.” Sami scholar Rauna Kuokkanen made a halcyon cry for the academy to “take responsibility” and indigenize. In her article in the *Journal of Curriculum Theorizing* from 2010, Kuokkanen intoned,

In the contemporary academy, there is very little awareness of indigenous epistemes beyond the occasional surface recognition of the existence of indigenous knowledge. What is more, the academy, in general, is very reluctant, in spite of its profession of knowledge, to expand its narrow and exclusionary epistemic foundations and, thus, to take its responsibilities in producing knowledge (p. 61–2).

Using the concept of responsibility to alter and therefore forward this reality toward the inclusion of Indigenous epistemes, Kuokkanen calls for the academy of higher learning to:

Do its homework pertaining to Indigenous epistemes…[as] part of the larger project of shifting the attention from common institutional approaches seeking to mainstream and “acclimatize” Indigenous students to the culture and convention of the academy to investigating the role of the academy with regard to other than its own foundational epistemes in its production and politics of knowledge (Kuokkanen, 2010, p. 61).

While the inclusion or exclusion of Indigenous epistemes of knowledge is beyond the scope of this article, I would like to mention that there seems to be another disconnect between signaling support for Indigeneity and implementing it. Other scholars recognize “Peace education and human rights education are not commonly used approaches in Indigenous education” (Huaman, 2011, p. 244), and while there are studies that seek to understand how peace education can be implemented in Indigenous culture (Makoni and Higgs, 2016) and how Indigenous notions of peace can factor in post-conflict development (Rahman et al., 2023) a technique for non-indigenous educators to bridge Indigenous values with Western Peace Education values, has yet to be explored. Indigenous education inherently embodies many of the principles of peace education. This is why, in my view, there needs to be an identifiable and operational roadmap for Pākehā to do more than understand worldview, epistemology and learning styles; a bicultural pedagogy is needed. The red flags of a potential bicultural “peace” pedagogy are shared by other Western-centric peace educations that “assume democracy, capitalism, individual human rights, and international law alone to be the universal foundations of a just world peace” (Kester et al., 2019, p. 10). At its base, a bicultural “peace” pedagogy would need to do more than seek to replace current Western-centric worldviews; it will need to “honor the treaty…[and] recognize the special status of Māori” and seek to articulate Indigenous pedagogy to peace pedagogy (Standish, 2019, p. 126); because a bicultural peace pedagogy would need to be both an instrument of decolonization and conflict transformation.

From my vantage point, this aim remains uninvestigated, and I hope to contribute a deeper view of this puzzle in this paper. My goal in this endeavor is to grow my own potential to share mātauranga Māori and share this bicultural peace pedagogy “methodology” with others. Along those lines, the proceeding examination will seek to respond to Ritchie's call in 1992 and elevate and amplify five aspects of Ritchie's “credo” relevant to peace pedagogy: *manaakitanga*-in all things lead with care for others; *putahi*, a unity of all things; *manatangata*- all persons deserve respect; *manawairua*-spirit pervades and must be respected; *whakakitenga*-there is more to know, do not imagine you fully understand (1992).

**Figure 1:**
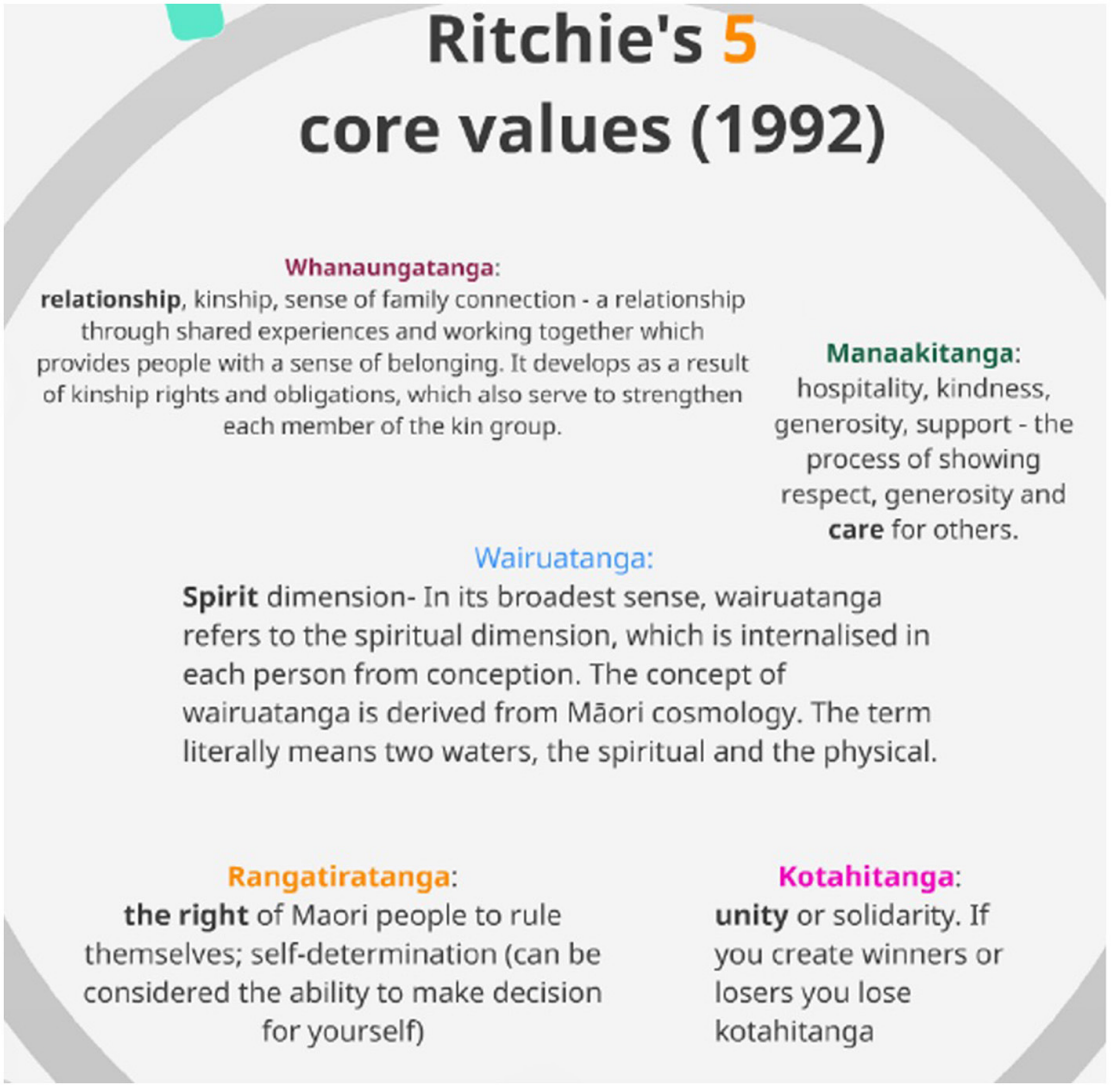
Richie's 5 core values of Ritchie (1992).

Biculturalism is defined in Aotearoa/New Zealand in relation to the 1840 Titriti o Waitangi/Treaty of Waitangi that formally recognized two cultural groups: the Indigenous Māori and settlers, the non-Indigenous, Pākehā (Ritchie, 1992). In North America, biculturalism can be interpreted as an awareness of non-white (previously termed “minority” culture) or Indigenous epistemes in the dominant colonial culture but also the “concept of navigating across worlds” (Darder, 1991; Schwartz and Ungerb, 2010, p. 26). Though considered both neutral (banal) and diverse (yet equal) in anthropological literature, culture can also be examined in relation to conflict (Giroux, 1981). Much more than a shared symbolic landscape of understanding, in multicultural spaces, culture must also be appreciated as a form of truth, a form of hegemony, and a form of power with the potential to subjugate and violently marginalize. Peace education, a field concerned with imparting skills, attitudes and knowledge that leads to non-violence and human flourishing, is an educational approach to the transformation of conflict that emerges from recognizing how we “learn” to be violent (Harris, 2004). But how would such an approach, designed to counter the violence of mainstream colonial education—violence against the self, others and society—align with initiatives that seek to decolonize the academy and unseat dominant settler/colonial institutional epistemes? This article seeks to begin to unravel that potential by considering a hypothetical constellation of Decolonizing Education, Indigenous Pedagogy and Peace Pedagogy in the context of Māoridom. In this report, I propose to offer key values (see Figure 1) of mātauranga Māori including wairuatanga, manaakitanga, kotahitanga, whanaungatanga, and rangatiratanga and upon this foundation, I will propose a complementary conjunction with Indigenous pedagogy and then peace pedagogy (Ritchie, 1992; Kanu, 2011; Harris, 1990).

My goal is to form a constellation of meaningful associations that seek to bridge cultural/pedagogical practice. The core of a bicultural peace pedagogy centers Indigeneity, this paper will introduce Ritchie's five Māori values to then conjunct them outward. In this proposal, Western peace pedagogy forms the fringe or margins of the pursuit of bicultural peace pedagogy and utilizes Kanu's 14 facets of Indigenous pedagogy as the interspace (Kanu, 2011).

## Decolonization

Colonization is “the imposition by a foreign power the direct rule over another people,” and decolonization is a process whereby places and peoples act to politically and culturally remove dominatory structures and mindsets derived from invader cultures (Betts, 2012, p. 1). Colonies were created to supply (largely) European powers with resources and labor fueling great wealth. In places where the colony became an outpost of the empire, such as Aotearoa/New Zealand, the form of invasion and occupation is described as “settler-colonization,” a form of colonization where the colonizer not only did not ever leave but where the settler culture displaced and sought to eradicate existing Indigenous culture (Jackson, 2019). Various social, political, economic, and cultural structures were imposed on Indigenous cultural groups that formally institutionalized settler culture—one such structure is schooling. While the scope of this paper does not permit a detailed exploration of global colonial education and its violent imposition upon Indigenous peoples, the trajectory of decolonizing education is pertinent to this article's aim of presenting an de-colonial construct regarding education. As Smith conveys, decolonization “is about centring our [Indigenous] concerns and world views...” (2012, p. 41), so decolonizing education is about centring Indigeneity in the learning space.

### Decolonizing education

Decolonizing pedagogies—distinct from critical, feminist, anti-racist, and humanist pedagogies—begin with the assumption that colonialism and imperialism are the center of our oppression. Although other pedagogical approaches can be integrated within the broader framework of de-colonization, a distinguishing feature of decolonizing pedagogies is their explicit engagement with the question of colonialism at all levels of education (Zavala, 2016, p. 156).

In places where Indigenous culture has been successful in fighting the “monocultural, melting-pot ideology of ethnic relations [involving] one language, [and] one education system,” decolonizing education has sought to Indigenize the curriculum (Ritchie, 1992, p. 9). This is a process that entails both unearthing the imperialist “culturalism” inherent in colonized education (one that is non-Indigenous, imposed, and mainstreamed in most countries in the world) and unraveling and reimagining the values and practices of “education” in the modern age to include Indigenous, minority and subjugated cultures (Darder, 1991; Battiste, 2013). Decolonizing education,

is a process of unpacking the keeper current in education: its powerful Eurocentric assumptions of education, its narratives of race and difference in curriculum and pedagogy, its establishing culturalism or cultural racism as a justification for the status quo, and the advocacy for Indigenous knowledge as a legitimate education topic (Battiste, 2013, p. 107).

It utilizes educative spaces to deconstruct, resist, revision and reinvigorate learning but it does so in largely mainstream schooling which means the vessel of education—schools—remain even if the content (curriculum) and teaching styles (pedagogy) may change (Standish, 2019).

Enduring colonial constructs in society are problematic, but future educations, however they manifest, continue to undergo transformations away from formal colonial-type schooling for reasons of philosophy and criticality, making the schooling of the past truly a thing of the “past” in most educative spaces. Mainstream pedagogues in ECE, primary and secondary schooling hardly walk up the aisle of the classroom with meter-long rods for discipline anymore. The teacher is not a nun, a priest, or a spinster, and the assignments are not solely about writing, reading, and arithmetic. And return to a pre-colonial pedagogy is impossible—traditional education was largely a facet of apprenticeship and gaining cultural knowledge to live in each society with others comprised of “wholistic [sic], lifelong and utilitarian” learning (Adeyemi and Adeyinka, 2003, p. 425; Delanty, 2018). Western colonial education was academic and literary, and although different, many Indigenous groups embraced such education and saw benefits in schooling their young as Western education was considered a pathway to opportunity (Mander et al., 2015).

Although Māori had whare wānanga (schools for teaching their own genealogy of knowledge), they wanted to send their children to the mission schools to access the Pākehā knowledge that produced large ships, powerful weapons and an amazing array of goods. Perhaps schooling would unlock the secret to this material wealth (Walker, 2016, p. 21).

What transpired afterward, however, was not simply “more” and “different” education but a utilization of education in the cultural colonialism^1^ of eradication. In religious schooling, in particular, the goal was not simply to benignly educate the next generation but to strip them of their cultural grounding, their values, their symbols, their considerations and, as a signifier of all three, their language (Regan, 2010; Battiste, 2013; Grande, 2015). In absolute genocide (both instrumental and cultural), the bodies of the Indigenous themselves were annihilated (Truth Reconciliation Commission of Canada, 2015).

“Education is not an element that can be detached from one civilization and borrowed by another. It is the concentrated epitome of a culture, and as such, it is inseparable from the form of that culture” (Marrou, 1982, xviii). To be educated in a colonized country, colonized people learn that *their* values, *their* stories, *their* ways of living, and *their* knowing are *less than*; because *their* language, *their* metaphysics and *their* mentalities are absent from the classroom and consequently unworthy (Smith, 2012). As Sullivan observed in 1999, “The issues surrounding values education and values in education need to be more extensively considered and debated; and that the cultural pluralist dimension, particularly in relation to issues in Maori education,” is needed (p. 205), but how? Analysis of the pitfalls have concluded, “…mainstream institutions could ever be adapted to meet the educational ambitions of Māori seems doubtful because the changes called for are too radical and the problems too institutionalized to be overcome easily. In addition, it seems unlikely that a kaupapa Māori philosophy would ever be adopted by a mainstream institution” (Hook, 2007, p. 9), but the work is still needed.

Decolonizing education involves unraveling this cultural supremacy and opening previously exclusive colonial “cultural” values in education. This is difficult and challenging as a complete return to pre-colonial Indigenous ways of knowing, believing and behaving is impossible (Bishop, 2003). Despite the hurdle, included in the decolonizing initiative is the attempt (more difficult in spaces with extreme culture loss) to resurrect fluency and creativity in Indigenous languages and to make present the lifeworld of a culture and a people whom the colonizers sought to initially erase or ultimately assimilate through education (Regan, 2010; Battiste and Youngblood, 2000; Cote-Meek, 2014).

An important part of decolonization education is the role of schools and teachers in acknowledging Indigenous knowledge and lifeways in “culturally appropriate” ways that contribute to educative gains for Indigenous learners (Black and Hachkowski, 2019, p. 1,094; Aseron et al., 2013). Such practices foreground and honor Indigenous ways of knowing and learning to provide a safe space for “culturally oriented border crossing” between Indigenous ways and Western education (Arnold, 2017, p. 476). Creating and supporting a bridge between these two worlds of education means ideally having teachers who can move between the Western and Indigenous worlds. In Aotearoa/New Zealand, that means a largely Pākehā teacher pool (about three-quarters of teachers are non-Māori) able to both understand and support non-Western students.^2^

### Posting a bicultural peace pedagogy

To upskill and educate us educators, it seems like we non-Māori all need to go back to school. With this intention in mind, this aptitude to “move between worlds,” this paper will compose and communicate a potential rubric of endeavor that permits border crossing as peace pedagogy. Indigenous peoples have been forced to border cross for centuries (as females have had to adopt the male gaze under male supremacy), so this work, the work proposed in consideration of a bicultural peace pedagogy, signals from the outset that as most teachers in Aotearoa/New Zealand are Pākehā the mahi (work) of bicultural peace pedagogy is the mahi of Pākehā. To facilitate this transformation, “more” is needed. While pedagogy has a role to play in decolonization as it has in alternative (read: non-Western/colonial) pedagogies that seek to recognize students holistically (a whole human: accessible/flexible/participatory/experiential approach) the mahi cannot begin without a fundamental understanding of culture.

As all education systems are “cultural artifacts” to change education, we need to change culture. In the next section, this paper will present a cultural core of the Māori world, followed by a brief exploration of an Indigenous pedagogy. These two facets of understanding comprise the center of a bicultural peace pedagogy, which is orbited afterward with facets of peace pedagogy to investigate the potential—the obstacles and opportunities—of a bicultural peace pedagogy.

#### Ritchie's credo

In 1992, James Ritchie sought to create a roadmap of interaction between Māori and Pākehā (Ritchie, 1992) and in the foundation of this principle to action was an appreciation of the five underlying or “core” values held by Māori. As a cultural outsider, Ritchie did not claim to have expert or intimate knowledge of these cultural values, but he considered that authentic biculturalism would mean Pākehā could not begin to do the “work” of decolonization without appreciating and centering the following beliefs and ethics:

*Wairuatanga*: spirit pervades and must be respected;

*Manaakitanga*: In all things, lead with care for others;

*Kotahitanga*: a unity of all things, to make something “less” diminishes all;

*Whanaungatanga*: relationship, kinship and sense of family connection; and,

*Rangatiratanga*: the right to self-rule, self-determination, and to make decisions for yourself.

These core values form a stratum of meaning underneath what it means to “be” Māori. To be Māori is to exist in spirit and care, together, connected and powerful. The disruption of colonial education reflected none of these values. Western education values the individual more than the group, “encourages individualism,” and rejects “conformity,” rewarding students for creativity and individual success (Aminuddin et al., 2010, p. 4).

In Māori epistemology,

Humans are born into the world with no knowledge. All knowledge emanates from the gods, who embedded it in the natural world to be discovered by humans. For this reason, the pursuit and transmission of knowledge was a sacred enterprise confined to whare wānanga (Walker, 2016, p. 21).

In Western education, knowledge is a commodity, something to be acquired and observed to be legitimate. Epistemology, or knowledge, goes back to Plato, who saw knowledge as a form of reality or truth and education as a platform for developing morality, character, abstract reasoning, skill, and strength (Gutek, 2001). Dozens of educational theorists, and hundreds of “mission” schools later, Western education is even more focused on acquiring capabilities, mentalities and credentials. Having examined Ritchie's core of Māoritanga and scratched the surface of Western Education, we will now turn to Indigenous pedagogy.

### Indigenous pedagogy

Indigenous pedagogy uses teaching and learning as a way to decolonize both learners and the structures of learning, i.e., schools, learning methods, and curriculum (Kanu, 2007, 2011; Battiste and Henderson, 2009; Castagno and Brayboy, 2008; Marker, 2006, 2011). Its purpose is to eradicate coloniality in schools, to counter anti-Indigeneity in society, revive Indigenous language and culture in settler colonial nations and support Indigenous people. In the *Handbook of Indigenous Education*, McKinley and Smith (2019) aver that,

Indigenous education was not always marginalized. Indigenous communities have always maintained and developed complex education systems. However, colonial invasion and exploitation have shattered Indigenous knowledges and ways of knowing, and as a result, the pieces have become scattered—destroyed, hidden, and other parts just waiting to be reconstructed (p. 1).

Despite Richie's call, this article recognizes that the mahi (work) of restoration inherent in Indigenous Pedagogy is not for Pākehā. It is for Indigenous peoples to rediscover, reconstruct, reconnect and restore their cultural ways of knowing, teaching and learning. For outsiders to decide and define, the content and form of Indigenous restoration is just one more form of colonial academics, cultural oppression and violence (Jackson, 2019). For this paper, what I had hoped to present was a pathway or bridge that relates to peace pedagogy. There are hundreds of different Indigenous groups worldwide and, equally, many different hapu and iwi within Aotearoa/New Zealand today, but I offer Yatta Kanu's rubric of Indigenous pedagogy to elevate common Indigenous pedagogies that work toward decolonization. This rubric is recognized by aboriginal scholars/practitioners and presented in the *Decolonizing Pedagogies Teacher Reference Booklet* developed by the Vancouver School Board in British Columbia, Canada (McGregor, 2012).

Having recognized that teachers, parents and learning community members need to understand what a decolonizing pedagogy entails and entertains, this rubric seeks to take fragments of understanding, scholarship and practice that make meaningful pedagogies of decolonization for teachers in the classroom to create positive learning spaces (McGregor, 2012). As this article seeks to investigate a potential framework for bridging Indigenous pedagogy and peace pedagogy, this forms the framework or foundation of this inquiry.

#### Peace pedagogy

Peace Pedagogy uses teaching and learning as a way to transform direct, structural and cultural forms of violence (Galtung, 1990). It is a core part of “peace education,” a field of Peace and Conflict Studies that seeks to recognize conflict/violence, transform conflict/violence non-violently and contribute to human thriving (Harris, 2004; Harris and Morrison, 2013). Earlier in this piece, the term “biculturalism” was used to signal an ability of “navigating across worlds” (Schwartz and Ungerb, 2010, p. 26). The two worlds this piece seeks to connect in the remainder of this report, to bridge and perhaps inhabit so that culture can permeate fitfully, are Indigenous and Peace Pedagogy. To do this, this paper has deliberately centered on Māoritanga and Indigenous pedagogy before seeking to apply facets of peace pedagogy.

Looking closely at the comparative table (see Table 2), it can be concluded that many pedagogical values that are present in Indigenous pedagogy^3^ are also present in peace pedagogy, specifically:

While many synchronicities exist, there are two notable gaps in Table 1, using field (land) visits outside of the colonized classroom (#4) is one, but importantly, and critically for our exploration of a potential “bicultural peace pedagogy” item (#3). “Learning scaffolds that support differing learning styles and incorporate Aboriginal content,” indicates a missing component of decolonizing *peace* pedagogy.

**Table 1:** Indigenous pedagogy (Kanu, 2011 as cited in McGregor, 2012, p. 7).

	**Indigenous pedagogy**
1.	Stories as a teaching method
2.	Sharing/talking circles
3.	Learning scaffolds that support differing learning styles incorporate aboriginal content.
4.	Field trips
5.	Guest speakers
6.	Activities that accommodate multiple learning styles
7.	Opportunities for student-driven decision-making and problem-solving
8.	Openness for students to speak honestly
9.	Encouraging students to listen to each other
10.	Sense of belonging in the learning space
11.	Students don't feel unsafe
12.	Teachers respect student silence
13.	Opportunities to counter stereotypes
14.	Help students explore themselves and their values.

### A bicultural peace pedagogy potential

**Figure 2:**
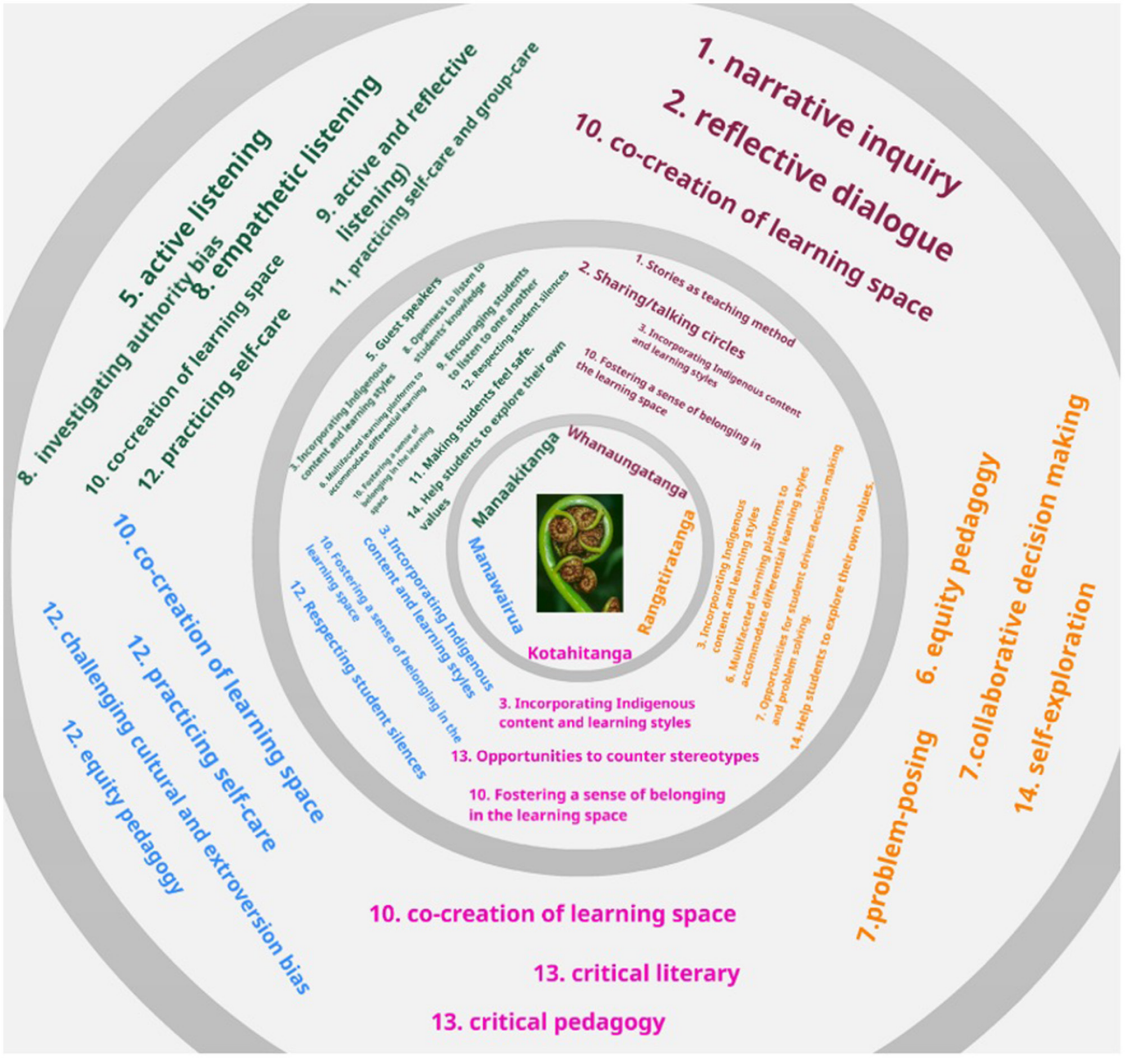
Māoritanga in Indigenous and peace pedagogies (author's collection).

Aligning peace pedagogies and Indigenous pedagogies while centering Richie's 5 Core Values of Ritchie (1992) creates the following graphic representation (See Figure 2).

Indigenous pedagogy (middle circle) can be seen to fully enmesh with the core of Māoritanga (inner circle), whereas the outer margins of peace pedagogy contain most but not all the inner circle values. That means that the capacity for peace pedagogy to carry out decolonization is limited by its lack of rooting in (in this instance) Indigenous Māori culture. Although not a perfect alignment, with 12 of 14 facets of Kanu's Indigenous pedagogy, an opportunity to decolonize via peace pedagogy exists (see Table 2). But impediments do too, and although the focus of this paper has been to investigate the potential for a bicultural peace pedagogy, further inquiry will need to investigate a fundamental orientation between Indigeneity and Western peace education that can be identified by closer inspection into culture, namely: the Individualism-Collectivism or *I-We* divide. Considered a facet of psychological culture relating to values that prioritize personal goals over the goals of the group, there is a fundamental (I would term “prime”) orientation in each—Western culture and Indigenous culture—that may be incompatible. Although critical peace education (Bajaj, 2008a) focuses on structural violence in society (the group), the *individual* is the undeniable instrument of peace education (Harris, 2004). The focus of using peace education is the transformation of the individual—even in interpersonal and inter-group dynamics—is prime (Harris and Morrison, 2013). This is in complete inversion to Indigenous ways that formulate the group as prime: “Whanaungatanga [the family] is the basic cement that holds things Māori together” and can be translated as *whanau* (family) *nga* (extended out) *tanga* (into relationship; Ritchie, 1992, p. 67).

**Table 2:** Aligning Indigenous and peace pedagogy.

	**Indigenous pedagogy**	**Peace pedagogy**
1.	Stories as a teaching method	Narrative pedagogy (Harris, 1990; Bekerman and Zembylas, 2012)
2.	Sharing/talking circles	Reflective dialogue (Harris, 1990; Jenkins and Jenkins, 2010; Reardon and Snauwaert, 2011)
3.	Learning scaffolds that support differing learning styles and incorporate aboriginal content	
4.	Field trips	
5.	Guest speakers	Active listening, (Harris, 1990, 2004; Harber and Sakade, 2009)
6.	Activities that accommodate multiple learning styles	Equity pedagogy (Ardizzone, 2002; Bajaj, 2008a; Bajaj and Hantzopoulos, 2016; Trifonas and Wright, 2013)
7.	Opportunities for student-driven decision-making and problem-solving	Problem-posing (Freire, 2003)
		Collaborative decision making (Harris, 1990; Lum, 2013; Jenkins and Jenkins, 2010)
8.	Openness for students to speak honestly	Empathetic listening (Zembylas, 2007; Noddings, 2012)
9.	Encouraging students to listen to each other	Active and reflective listening (Harris, 1990; Jenkins, 2008)
10.	Sense of belonging in the learning space	Co-creation of learning space (Freire, 2003; Jenkins, 2008)
11.	Students don't feel unsafe	Practicing self-care and group-care (Noddings, 2012; Standish and Joyce, 2018)
12.	Teachers respect student silence	Practicing self-care, equity pedagogy (Jenkins, 2015; Standish and Joyce, 2018)
13.	Opportunities to counter stereotypes	Critical literary and critical pedagogy (Giroux, 1988; Reardon, 1988; Jenkins, 2015; Standish, 2015)
14.	Help students explore themselves and their values	Self-exploration and empathy building (Standish and Joyce, 2018; Bajaj, 2008b)

## Conclusions

This article has offered an appreciation of decolonization in education, positing a bicultural pedagogy as peace pedagogy. It offered conceptualizations of Indigenous pedagogy and peace pedagogy to evaluate the potential of bicultural peace pedagogy as a decolonizing education. Though “fitting pieces” appears opportune, an obstacle of orientation must be appreciated to comprehend compatibility (or lack of it) based on culture. The roots of Western and Indigenous culture do not align; that is a definite challenge, which means that Indigenous pedagogy and peace pedagogy as a construct of Western peace education also do not align. Earlier in this paper it was mentioned that “as all education systems are” “cultural artifacts in order to change education, we need to change culture.” As the root of violence in colonial education is Western culture, Western culture in education may need to change.

## Author's note

Dr. Katerina Standish is Associate Professor of Global and International Studies and Vice Provost Graduate and Postdoctoral Studies at the University of Northern British Columbia, Canada. Formally at the University of Otago, in Aotearoa/New Zealand, she is the author/editor of *Perspectives on Justice, Indigeneity, Gender, and Security in Human Rights Research, Suicide Through a Peacebuilding Lens, The Palgrave Handbook of Positive Peace* and *Cultural Violence in the Classroom*.

## Data Availability

The original contributions presented in the study are included in the article/supplementary material, further inquiries can be directed to the corresponding author.

## Footnotes

^1^“The term cultural colonialism refers to the extension of colonial state power through cultural knowledge, activities, and institutions (particularly education and media) or the systematic subordination of one conceptual framework or cultural identity over others” (Amsler, 2007, para 1).

^2^The most recent teacher census was in 2004: teachers were only 10% Māori and 79% European/Pākehā: https://www.educationcounts.govt.nz/publications/schooling/teacher_census.

^3^Missing values include 3: *Learning scaffolds that support differing learning styles and incorporate Aboriginal content* and 4: *Field Trips*.
